# Dhurrin: a potential endogenous nitrogen turnover source for early seedling growth in sorghum

**DOI:** 10.3389/fpls.2025.1558712

**Published:** 2025-05-08

**Authors:** Yves Emendack, Jacobo Sanchez, Haydee Laza

**Affiliations:** ^1^ Cropping Systems Research Laboratory, United States Department of Agriculture - Agricultural Research Service (USDA-ARS), Lubbock, TX, United States; ^2^ Davis College of Agricultural Sciences and Natural Resources, Texas Tech University, Lubbock, TX, United States

**Keywords:** dhurrin, nitrogen, recycling, turnover, seedling, metabolism, sugars

## Abstract

Dhurrin is a cyanogenic glucoside found in all vegetative tissues of *Sorghum bicolor*, functioning as a herbivore repellent, antifungal agent, osmoprotectant, and nitrogen (N) storage. Dhurrin concentration is usually highest in young seedlings, where it rapidly accumulates following germination, after which its biosynthesis decreases and its turnover increases as the seedling ages. To avoid prussic acid poisoning from dhurrin catabolism in cattle grazing or foraging on sorghum, numerous research studies now focus on breeding for dhurrin-free or acyanogenic sorghum using EMS (Ethyl methanesulfonate) mutants with a non-functional dhurrin biosynthetic pathway. However, there has been limited and conflicting research investigating the role dhurrin plays as a potential nitrogen source in sorghum’s early seedling growth, especially under N deficiency. It is plausible that the presence of background mutations in dhurrin-free sorghum mutants could mask or confound how the absence of dhurrin affects early seedling growth. Using a naturally occurring (non-mutant) ultra-low dhurrin genotype and known low and high dhurrin genotypes, the current research investigated the importance of dhurrin as a potential endogenous nitrogen source for early seedling growth in simulated non-marginal (N-available) and marginal (N-deficient) media. Dhurrin was implicated to be an N source for seedling growth from 8 to 13 days after planting under deficient N conditions. In N-deficient media at 13 days after planting, high-dhurrin-level genotypes accumulated more seedling fresh shoot biomass than low-dhurrin-level genotypes. Thus, while acyanogenic sorghum will be beneficial in expanding sorghum’s economic value, the use of dhurrin knock-out mutants can prove problematic since the complete lack of dhurrin may affect field germination and stand establishment, particularly under N-deficient or low-N-input conditions.

## 1 Introduction

Sorghum is an important cereal crop grown worldwide for both human and animal consumption and is widely known for its ability to grow in marginal soils and tolerate drought and high temperatures. Sorghum is cyanogenic, with the cyanogenic glucoside, called dhurrin [(S)-p-hydroxymandelonitrile-*β*-D-glucopyranoside], found in all its vegetative tissues. The biological functions of cyanogenic glucosides include herbivore repellent (Jones, 1998; Gleadow and Woodrow, 2002); antifungal activity (Siebert et al., 1996); nitrogen (N) transport, turnover, and storage (Kongsawadworakul et al., 2009; Møller, 2010); and osmo-protectant properties (Burke et al., 2015). Dhurrin also serves as a deterrent to insect feeding (Tattersall et al., 2001; Krothapalli et al., 2013); plays a role in the storage, transport, and partitioning of nitrogen in the plant, increasing nitrogen use efficiency (Blomstedt et al., 2018; Rosati et al., 2019); and contributes to post-flowering drought tolerance (Burke et al., 2013; Hayes et al., 2015; Varoquaux et al., 2019). Dhurrin concentration is highest in ontogenetically young sorghum tissues, rapidly increasing following germination, after which its biosynthesis decreases and turnover increases, leading to a decrease in its concentration as the seedling ages (Adewusi, 1990; Busk and Møller, 2002). Dhurrin content is highest in the tip of young seedlings, reaching 6% of the dry weight (Akazawa et al., 1960; Halkier and Møller, 1989). The content of dhurrin in the later stages of plant growth and development depends highly on growth conditions and genetic background, increasing under N fertilization, drought, and frost (Busk and Møller, 2002; Strickland et al., 2017; Emendack et al., 2018).

**Figure 1:**
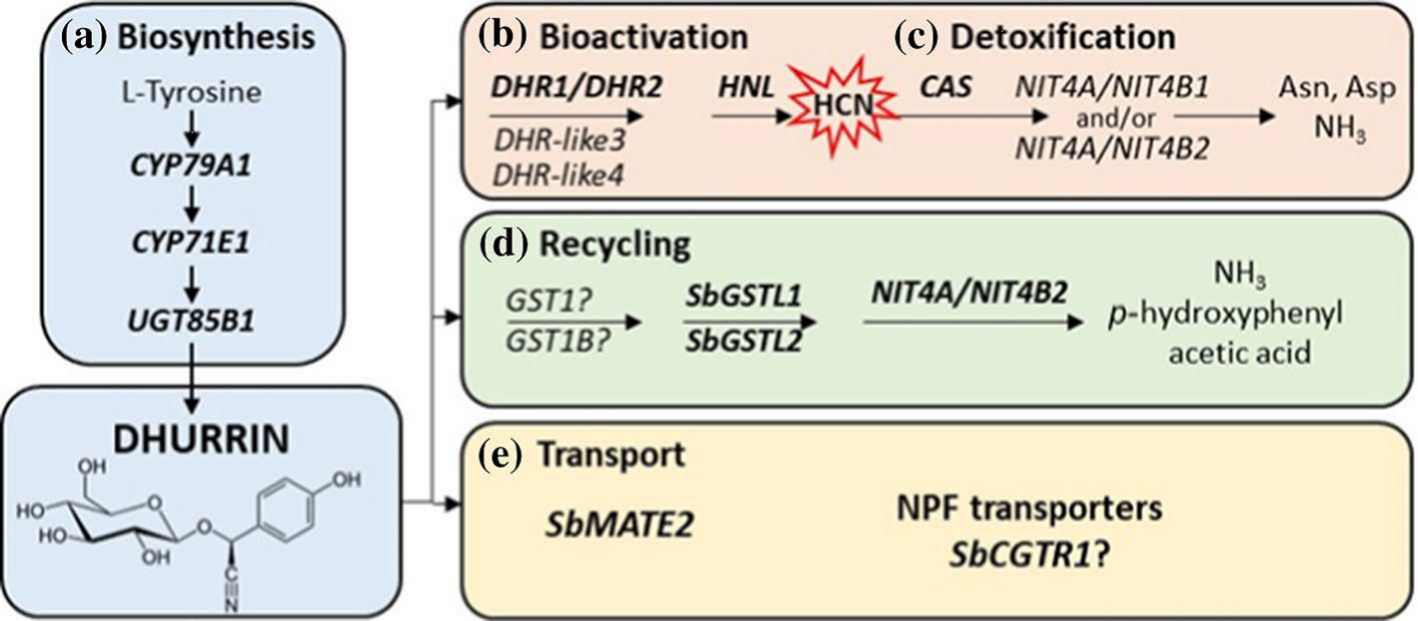
Scheme illustrating the synthesis and turnover/recycling of dhurrin in *Sorghum bicolor* [Gleadow et al. (2021): https://doi.org/10.1007/s00425-021-03774-2]. Overview of the genes involved in dhurrin biosynthesis, recycling, transport, bioactivation and in prevention of auto-toxicity. **(a)** Dhurrin is synthesised from tyrosine by the sequential action of three key enzymes. **(b)** Bioactivation of dhurrin to release a hydrogen cyanide bomb occurs upon tissue disruption by the action of specific β-glucosidases (DHR) and an α-hydroxynitrile lyase (HNL). **(c)** Auto-toxicity is prevented by β-cyanoalanine synthase and nitrilases retrieving the nitrogen of the hydrogen cyanide for production of amino acids and ammonia. **(d)** Endogenous recycling pathway mediated by glutathione transferases and a heteromeric NIT4A/NIT4B2 nitrilase resulting in formation of ammonia and p-hydroxyphenylacetic acid without the release of HCN. **(e)** Dhurrin synthesis occurs in the cytosol and two potential genes, SbMATE2 and SbCGTR1, may be involved in dhurrin transport to sites of storage within the cell or to other parts of the plant. Asn asparagine; Asp aspartic acid; DHR dhurrinase; NH3, ammonia. Reproduced by permission under a Creative Commons Attribution 4.0 International License (https://creativecommons.org/licenses/by/4.0/), which permits use, sharing, adaptation, distribution and reproduction in any medium or format.

Dhurrin degradation involves various enzymatic pathways (Gleadow et al., 2021, **Figure 1**). The first pathway is a toxic catabolic process (cyanogenesis) in which dhurrin is catabolized by the enzyme *dhurrinase*, rapidly generating toxic hydrogen cyanide (HCN) (Kojima et al., 1979; Jørgensen et al., 2005; Zagrobelny et al., 2008; Morant et al., 2008), which can be detoxified to either L-Asparagine or L-Aspartic acid and ammonia (NH_3_) as end products. The second is a non-toxic catabolic process that is termed the recycling or alternate turnover pathway, in which an unknown enzyme (which could be glutathione S-transferase) catalyzes the conversion of dhurrin to *p*-hydroxyphenylacetic acid and NH_3_, a nitrogen source (Adewusi, 1990; Piotrowski et al., 2001; Busk and Møller, 2002; Nielsen et al., 2016; Bjarnholt et al., 2018). The first by-product in either the catabolic or alternate turnover pathways of dhurrin is the simple soluble sugar glucose.

Sugars are the major reserve in seeds (Bewlay and Black, 1994) and are at maximum during germination, mobilized to various tissues (Smith, 1967) in the form of sucrose, fructose, and glucose, which are readily transportable and required for growth (Mayer and Poljakoff-Mayber, 1975). As a protection mechanism, plants under stress will convert the available simple sugar glucose to its complex storage form, sucrose (Rosa et al., 2009) or its isomeric form, fructose. Many studies have demonstrated that exogenous application of glucose resulted in a delay in germination, while sucrose is considered a major carbohydrate transport form upon seed germination and seedling development (Xu et al., 2010). Sorghum grain is low in fermentable oligosaccharides, disaccharides, monosaccharides, and polyols (FODMAPs). Oligo-or polysaccharides such as fructans consist of short chains of fructose units and a terminal glucose molecule. Fructans are the most abundant carbohydrate reserve in the vegetative organs in cereal grains (Pollock and Cairns, 1991) and are generally synthesized from sucrose (Edelman and Jeeeord, 1968; Cairns and Pollock, 1988). Fructan metabolism appears to be active early in seed development and late in germination, and has been associated with seedling growth (Pollock and Lloyd, 1994).

Due to its climatic resilience and being a main staple for subsistence in developing areas of the world, sorghum is usually grown in marginal lands where pre-plant N fertilization is often lacking (Adam et al., 2020). Additionally, the importance of dhurrin during sorghum seedling growth has been described (Sohail et al., 2020). Therefore, the plausible turnover of dhurrin as a potential nitrogen source for seedling growth under N-deficient environments is worth investigating. Research by Adewusi (1990) showed that dhurrin content and the cyanide potential (via catabolism) peaked 4 days after germination, at which point the rate of dhurrin synthesis and breakdown were equal; thereafter, it declined with plant age. Newton et al. (1980) reported that during early seedling development in sorghum, the endosperm sugar content continuously depletes while dhurrin content increases, with approximately 75% of the endosperm being depleted by 8 days after planting (4–5 days after emergence), at which point dhurrin content is at its maximum. Thus, in the absence of inorganic nitrogen and with seed endosperm resources depleted, early seedling growth could be slowed, and it begs the question whether dhurrin could be used as an N source to sustain growth. Furthermore, could the dhurrin levels determined from the leaves of matured plants (Burke et al., 2013) be a significant determinant in early seedling growth performance? Haskins et al. (1987), using high and low cyanide potential sorghum genotypes, reported that both genotypes had similarly high cyanide potential at the seedling stage, but when the upper leaves of field-grown plants were tested late in the vegetative stage, there was a markedly higher cyanide potential in favor of the high cyanide genotypes.

There is limited and conflicting research investigating the role dhurrin plays in sorghum early seedling growth in combination with other secondary metabolites such as glucose, fructose, sucrose, and fructans. Using mutagenized sorghum seeds, Gruss et al. (2022) suggested that dhurrin-free or acyanogenic *Tx623 bmr6 cyp79a1* mutant plants grew more rapidly than the wild-type but were more susceptible to fall armyworm feeding. However, other research using total cyanide deficient mutants (*tcd1*) showed a reduction in growth during germination and early seedling growth (Montini et al., 2020; Blomstedt, 2012; Sohail et al., 2020). Acyanogenic sorghum mutants have been developed through mutation in the catabolic enzyme SbEMS932 *dhr2-1* (Krothapalli et al., 2013) and the biosynthetic enzyme SbEMS2447 *cyp79a1* (Blomstedt, 2012; Tuinstra et al., 2016). It is plausible that the presence of background mutations in dhurrin-free sorghum mutants could mask or confound how the absence of dhurrin in these seedlings affects their early growth response. Using a naturally occurring (non-mutant) ultra-low dhurrin genotype and known low and high dhurrin genotypes (Burke et al., 2013; Emendack et al., 2018), the current research investigated the importance of dhurrin as a potential endogenous nitrogen source for early seedling growth in simulated non-marginal (N-available) and marginal soil (N-deficient) media.

## 2 Materials and methods

### 2.1 Experimental design

Six sorghum genotypes (**Table 1**) with known levels of leaf dhurrin content determined from the leaves of mature plants (Burke et al., 2013), were evaluated under two growth substrates [Professional potting mix Sunshine Mix 1 (Sungro Horticulture, Agwam, MA) and play sand (Quikrete, Atlanta, GA)] and two fertility regimes [Water soluble Peter^®^ Excel fertilizer (Everris NA Inc., Dublin, OH) and de-ionized water] combinations for early seedling growth performance. The Sunshine Mix 1 potting soil is a blend offering a good balance of peat moss, coarse perlite, vermiculture, major and minor nutrients, wetting agent, and dolomitic lime, making it a reliable soil media that professional growers use to grow a wide variety of crops.

**Table 1:** Sorghum genotypes with varying levels of dhurrin content used in the experiment.

Line	Source	^Ψ^Dhurrin level
BTX642	USDA-ARS, Lubbock	High
SC1154-14E	Sorghum Conversion Panel, Ethiopia	High
1790E	USDA-ARS, Lubbock	High
BTX623	USDA-ARS, Lubbock	Low
TX7000	USDA-ARS, Lubbock	Low
SC1506	Sorghum Conversion Panel, Mali	^Φ^Non-detectable

^Ψ^Based on measured dhurrin content from the second penultimate leaf at the flowering stage using HPLC analysis. ^Φ^Non-detectable from the second penultimate leaf at the flowering stage using HPLC analysis (Emendack et al., 2018). SCP, sorghum conversion panel.

Two experiments were conducted concurrently under controlled polyhouse conditions at the USDA–ARS laboratory in Lubbock, Texas. The Peter^®^ Excel water soluble fertilizer contained 15% total nitrogen, with 1.1% ammonia, 11.8% nitrate, and 2.1% urea nitrogen. Four substrate-fertilization combination treatments (T) were used: potting mix irrigated with N-fertilizer (soil +N; T1), potting mix irrigated with deionized water (soil -N; T2), play sand irrigated with N-fertilizer (sand +N; T3), and play sand irrigated with deionized water (sand -N; T4). Experiments were conducted in a polyhouse set at 28/20˚ C, day/night temperatures, 55% relative humidity, and 12/12-h day/night cycle using 400-W high-pressure sodium GE grow lightbulbs as supplemental lighting. In total, 32 seeds per genotype were sown in 32-insert jumbo plastic trays for each substrate-fertilization combination. Trays were monitored daily and sub-irrigated to prevent drying. The same seed-sowing and seedling-handling practices were applied in all experiments. In all experiments, the trays were randomized on greenhouse benches with four replicated trays per genotype per substrate-fertilization combination.

### 2.2 Seedling growth characteristics

Total above-ground shoot biomass (clipped at the crown) of 10 randomly selected representative seedlings, from each replicated tray, was harvested at 8 and 13 days after planting (DAP), equivalent to 5 and 10 days after emergence, respectively. Seedling length was measured from the base of the shoot to the tip of the youngest leaf. Each seedling was immediately weighed, carefully folded into a 2.0 ml microcentrifuge secured-lock tubes (Eppendorf, SafeLock^®^, Hamburg, Germany), avoiding crushing, placed on crushed ice, and transferred to -80˚ C storage until further analysis. HPLC analysis was performed to determine shoot concentrations of dhurrin and sugars (glucose, fructose, sucrose, and fructans). Hundred seed weight was determined by averaging the weights of three sets of 100 seeds per sorghum genotype.

### 2.3 HPLC analyses of dhurrin and sugars

Dhurrin and sugars were extracted from fresh shoot tissue in 1 ml of 80% ethanol. Briefly, samples were vigorously vortexed upon the addition of the ethanol and incubated at 60°C for 60 minutes on a Labnet AccuBlock™ digital dry bath heating block (Labnet International, Cary, NC). The extract was centrifuged at 10,000 rpm for 10 minutes, and 200μL of supernatant was transferred into a clean 2.0 mL microcentrifuge tube. The supernatant was then dried by vacuum centrifugation in a CentriVap system (Labconco Corporation, St. Louis, MO) set to a low drying rate. Dry extracts were re-suspended in 400µL HPLC-grade water (Fisher Scientific™, Hampton, NH) by placing a zinc-plated metal bead (Daisy^®^ BBs, Daisy Outdoor Products, Rogers, AK) in the 2.0 mL microcentrifuge tube containing the dried extract and mix-shaking for 1 minute at 30 cycles s^-1^ on a TissueLyzer^®^ mill (Qiagen, Hilden, Germany). Samples were then centrifuged at 11,000 rpm for 15 minutes to pelletize any particulates and 75µl of supernatant was carefully pipetted into properly labeled vials for HPLC analysis. Sugar separation analysis was performed using a 100 x 7.8-mm Rezex™ RCM Monosaccharide Ca^2+^ (8%) ion-exchange column (Phenomenex^®^, Torrance, CA) heated to 85°C and HPLC-grade water (Fisher Scientific™, Hampton, NH) as the mobile phase with a flow rate of 0.525 ml min^-1^. A Prominence Series HPLC system fitted with an evaporative light scattering detector (Shimadzu North America, Carlsbad, CA) was used for data collection and analysis. The presence of dhurrin and other sugars was determined based on their retention times in comparison to their corresponding known sugar standards. Dhurrin and sugar amounts (microgram - µg) were calculated by use of a log-log linear calibration curve of known dhurrin and sugar amounts (microgram - µg) injected into the HPLC system for analysis. The calibration curve was prepared from the following injected amounts of dhurrin/sugars: 7 µg, 5 µg, 3 µg, 1 µg, and 0.5 µg, and their respective peak heights. A log-log linear fit was fitted through the data with an R^2^ of 0.99933, a slope of 1.48769, and average deviation of the standards was 2.26%. The final concentration of each sugar and dhurrin was then determined by dividing the calculated amount (µg) in the injected sample by the equivalent fresh weight (milligram – mg) of the injected sample.

### 2.4 Statistical analyses

Data were analyzed using SPSS 22.0 (SPSS Inc., USA) and JMP 16 (SAS Institute). Multivariate analysis of variance was used to identify significant interactions of soil-fertilization combination treatments on fresh shoot weight, shoot length, dhurrin, glucose, fructose, and sucrose, based on Wilk’s Lambda test and its associated significant level expressed in relative partial Eta squared (η^2^). Partial η^2^ represents the proportion of the variance in the dependent variable(s) that can be explained by the independent variable(s). The Bonferroni adjustment was applied where necessary to avoid type I errors. Tukey’s honestly significant difference (HSD) test and Student *t*-tests separated means between and within genotypes for a given treatment. Pearson’s correlation matrix was used to identify correlations between parameters. Observed trends in the ranking of genotypes were based on the Tukey–Kramer HSD connecting letters ranking report for the assessed parameter. Significance was stated at *p ≤ 0.05* or *p ≤ 0.*01 where applicable. Percent change (%Δ) was used for within-genotype changes in assessed parameters from 8 to 10 days after planting as:


$$\%\Delta =\left({\text{X}}_{\text{T}13}–{\text{X}}_{\text{T}8}\right)/{\text{X}}_{\text{T}8}*100,$$

where X represents the value of the assessed parameter for specific treatment T at 8 and 13 days after planting.

## 3 Results

Observed variations in assessed parameters were consistently similar for the two experiments, so data were pooled together for multivariate analyses. Reported Partial Eta Square (η^2^) represents the proportion of the variance in the dependent variables and their interactions that the independent variables can explain. Using Cohen (1988), η^2^≤1% is considered a small effect, 1≤η^2^≤6% is considered a medium effect, and η^2^≥14% is considered a large effect.

The independent variables, seedling age (days after planting, D), substrate-fertility combination treatment (treatment, T), and genetics (genotypes, L) had effects on the observed variations in the primary (glucose, fructose, and sucrose) and secondary (dhurrin) metabolites (**Table 2**). Variation in dhurrin content was largely due to genetics (52.5%), followed by treatment x age interaction (33.4%), treatment applied (30.8%), and treatment and genetics interaction (29.0%). Only 9.4% of the variation in the dhurrin content was due to seedling age.

**Table 2:** Partial Eta Square (η^2^) values for the multivariate interactive effects of the independent variables (days after planting, D; treatment combination, T; and genotypes, L) on sugar concentration [dhurrin, Dhu (μg/mg); glucose, Glu (μg/mg); fructose, Fru (μg/mg); sucrose, Suc (μg/mg); and fructans, Ftn (peak value)] and seedling growth parameters [fresh shoot length, FSL (cm); and fresh shoot weight, FSW (mg)].

	Dhu	Glu	Fru	Suc	Ftn	FSL	FSW
………………………………….η^2^ (%) ….……………………….							
Days after planting (D)	9.4	23.2	3.8	74.0	16.5	80.1	80.2
Treatment (T)	30.8	57.0	37.1	65.3	74.6	73.1	80.2
Genotypes (L)	52.5	35.0	35	16.8	44.7	61.0	65.4
D x T	33.4	3.8	7.1	37.2	20.7	37.1	61.1
D x L	11.3	4.2	8.4	10.1	13.1	9.2	24.4
T x L	29.0	6.9	8.7	19.6	35.3	45.0	32.0
D x T x L	9.8	9.2	8.0	25.3	11.8	8.7	19.0
Mean	4.19	4.23	2.32	3.74	125,838^ϕ^	13.43	212.79
*± S.E.*	*0.47*	*0.67*	*0.39*	*0.64*	*42,950*	*0.58*	*17.17*

^ϕ^ Raw peak signal values from HPLC analyses; *S.E.* is standard error.

Generally, the variations in growth parameters were largely due to seedling age, treatment, and genetics. Variation in fresh shoot weight was equally (80.2%) due to seedling age and treatment applied, followed by genetics (65.4%), and age and treatment interaction (61.1%). Variation in shoot length was primarily due to seedling age (80.1%), and then treatment applied (73.1%), genetics (61.0%), and treatment and genetics interaction (45.0%). Only fresh shoot weight was largely affected (19.0% to 82.0%) by all the independent variables and their interactive effects.

The interaction of seedling age, treatment applied, and genetics had a large effect only on sucrose content (25.3%) and fresh shoot biomass (19.0%).

### 3.1 Early seedling growth

Early seedling growth and vigor depend on the availability of seed endosperm resources, 75% of which is depleted at 8 days after planting (Newton et al., 1980). In the absence of external sources of inorganic nitrogen or other remediations, seedling growth drastically slows or completely stops, which may result in unhealthy seedlings, necrosis, and seedling death.

Early seedling growth, based on fresh shoot length and fresh shoot weight, was evaluated at 8 and 13 days after planting across the four substrate-fertility treatment combinations.

**Figure 2:**
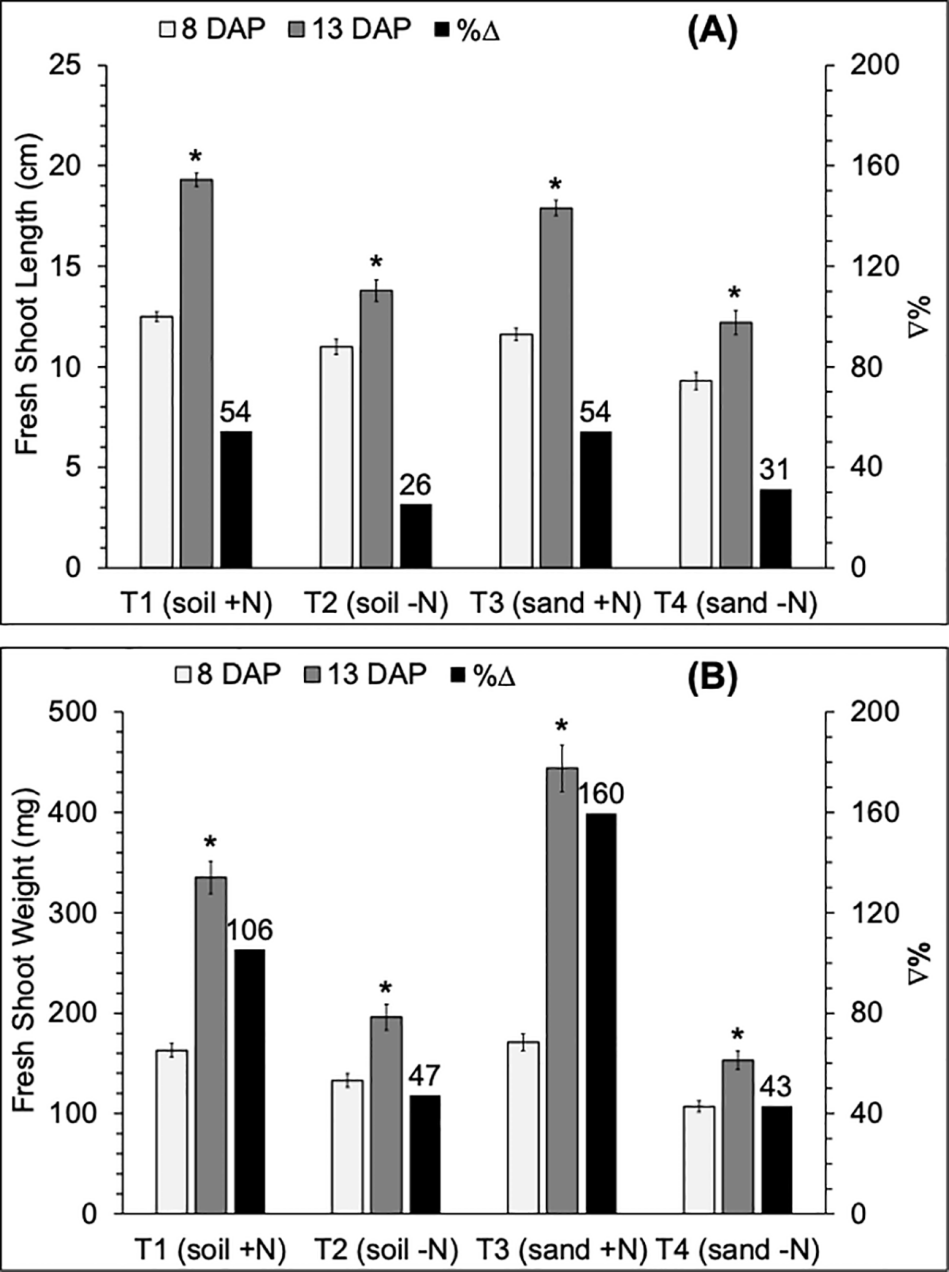
Variation (white and gray bars) and percent change (%Δ; black bar) of mean values of **(A)** fresh shoot length (FSL, cm) and **(B)** fresh shoot weight (FSW, mg) of 8 and 13 DAP sorghum seedlings grown in four substrate-fertility treatments under controlled conditions in a polyhouse. T1 is potting soil irrigated with N-fertilizer (soil +N), T2 is potting soil irrigated with deionized water (soil -N), T3 is play sand irrigated with N-fertilizer (sand +N), and T4 is play sand irrigated with deionized water (sand -N). *Indicates a significant difference (*p=0.*05) between DAP. Error bars represent the standard error of the mean.

Seedling length and weight significantly increased from 8 to 13 days after planting (DAP) irrespective of treatments (**Figure 2**). For both parameters, the variation in %Δ from 8 to 13 DAP was based on treatment. Percent increases in seedling growth parameters from 8 to 13 DAP were higher in N-available treatments [T1 (soil+N): FSL, 54%; FSW, 106%; and T3 (sand+N): FSL, 54%; FSW, 160%] compared to N-absence treatments [T2 (soil-N): FSL, 26%; FSW, 47%; and T4 (sand-N): FSL, 31%; FSW, 43%]. Seedlings grown under N-absence treatments (T2 and T4) were consistently shorter (**Figure 2A**) and weighed less (**Figure 2B**), compared to seedlings under N-available treatments (T1 and T3), irrespective of seedling age, significantly so at 13 DAP.

Under N availability at 13 DAP, seedling biomass was significantly higher in T3 (444 g) than in T1 (335 g) (**Table 3**). Under N absence at 13 DAP, seedling biomass was significantly lower in the T4 (153 g) compared to the T2 (196 g) treatment. In the absence of nitrogen, seedling length was significantly shorter in the T4 (9.3 cm) compared to the T2 (11.0 cm) treatment only at 8 DAP. Under N availability, seedling length was not significantly different between T1 (12.5 and 19.3 cm) and T3 (11.6 and 17.9cm) at 8 and 13 DAP, respectively.

**Table 3:** Variation of mean values of fresh shoot length (FSL, cm) and fresh shoot weight (FSW, mg) of 8 and 13 DAP sorghum seedlings grown in four substrate-fertility treatments under controlled conditions in a polyhouse.

Treatments	Fresh shoot length (cm)		Fresh shoot weight (mg)	
	8 DAP	13 DAP	8 DAP	13 DAP
T1	12.5 ± *0.24* A	**19.3** ± *0.33* A	163 ± *6.89* A	**335** ± *16.21* B
T2	11.0 ± *0.38* B	**13.8** ± *0.54* B	133 ± *6.78* B	**196** ± *12.90* C
T3	11.6 ± *0.31* AB	**17.9** ± *0.39* A	171 ± *8.34* A	**444** ± *23.17* A
T4	9.3 ± *0.44* C	**12.2** ± *0.60* B	107 ± *5.47* C	**153** ± *9.07* D
Mean ± *S.E.*	11.1 ± *0.20*	15.8 ± *0.35*	144 ± *4.15*	282 ± *13.24*

T1 is potting soil mix irrigated with N-fertilizer (soil+N), T2 is potting soil mix irrigated with deionized water (soil-N), T3 is play sand irrigated with N-fertilizer (sand+N), and T4 is play sand irrigated with deionized water (sand-N).Treatments with different mean value letter A, B, C…, for a given growth parameter (FSL or FSW) at a given seedling age (8 or 13 DAP) are significantly (*p=0.05*) different. *S.E.* is standard error of the mean. Bold numbers indicate a significant difference between 8 and 13 DAP for the given growth parameter under the specific treatment.

**Figure 3:**
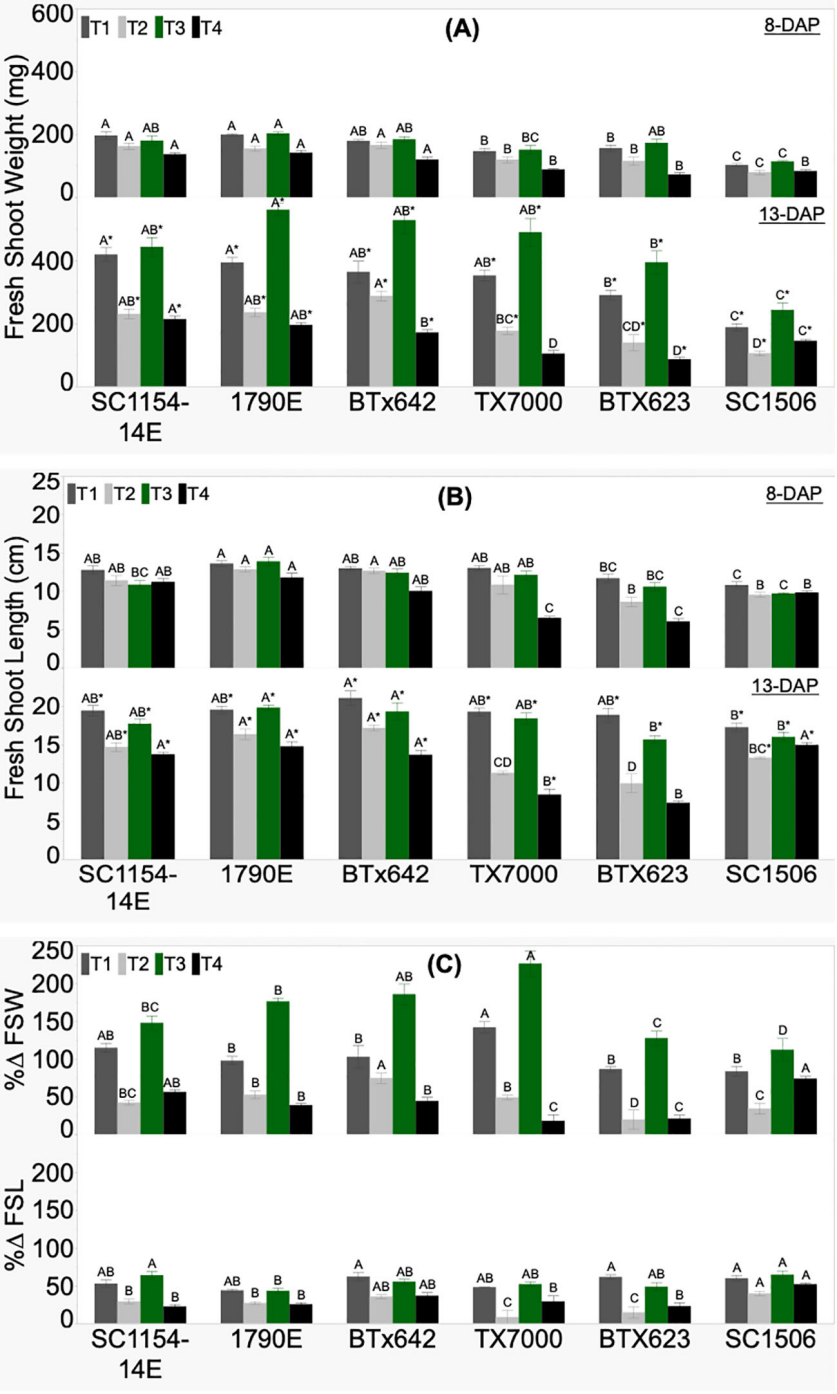
Genotypic variation of **(A)** fresh shoot weight (FSW), **(B)** fresh shoot length (FSL) at 8 and 13 days after planting (DAP), and **(C)** the percent change (%Δ) between DAP for FSW and FSL, of sorghum seedlings grown in four substrate-fertility treatments (T1, T2, T3, and T4). Bars with different letters A, B, C for similar treatment at a given DAP are significantly different. An asterisk (*) on a letter indicates a significant difference between DAP for the given genotype under the specific treatment. High-dhurrin genotypes: SC1154-14E, 1790E, BTx642; Low-dhurrin genotypes: TX7000, BTx623, SC1506.

Genotypic variability in FSW, FSL, and their respective %Δ was significant across treatments (**Figure 3**). Fresh shoot weight at 8 DAP showed a decreasing trend from high-dhurrin level genotypes (SC1154-14E, 1790E, and BTX642) to low-dhurrin genotypes (TX7000, BTX623, and SC1506) across all treatments, except under sand+N (T3), where BTX623 and TX7000 had similar FSW as the high dhurrin genotypes (**Figure 3A**). At 13 DAP, all high-dhurrin genotypes accumulated significantly higher fresh biomass than all low-dhurrin genotypes under sand-N (T4) only. FSW significantly increased from 8 to 13 DAP irrespective of genotype or treatment. Percent change for fresh biomass (%ΔFSW) was positive and significantly different across genotypes irrespective of treatment (**Figure 3C**). Only in T4 did all high-dhurrin genotypes show a higher %ΔFSW than all low-dhurrin genotypes, except SC1506.

FSL at 8 DAP did not show a clear trend from high-to-low dhurrin genotypes irrespective of treatment (**Figure 3B**). However, for T4 (sand-N) at 8 DAP, high dhurrin genotypes were significantly taller than low dhurrin genotypes except for the ultra-low dhurrin genotype SC-1506, which showed similar shoot length to the high dhurrin genotypes. At 13 DAP, in soil-N (T2) and sand-N (T4) treatments, all high-dhurrin genotypes had significantly higher FSL than all low-dhurrin genotypes, except SC1506. FSL significantly increased from 8 to 13 DAP irrespective of genotype or treatment. Percent change for shoot length (%ΔFSL) was positive and significantly different across genotypes irrespective of treatment (**Figure 3C**). Only in soil-N (T2) treatment did all high-dhurrin genotypes showed a higher %ΔFSL than all low dhurrin genotypes, except SC1506.

### 3.2 Dhurrin and secondary sugar metabolites

The first by-product in either the catabolic or alternate turnover pathways of dhurrin is the simple soluble sugar glucose (**Figure 1**). Sugars are the major carbon reserves in seeds (Bewlay and Black, 1994) and are at maximum during germination, mobilized to various tissues (Smith, 1967) in the form of sucrose, fructose, glucose, and fructans, which are readily transportable and required for growth.

#### 3.2.1 Dhurrin and glucose content

**Figure 4:**
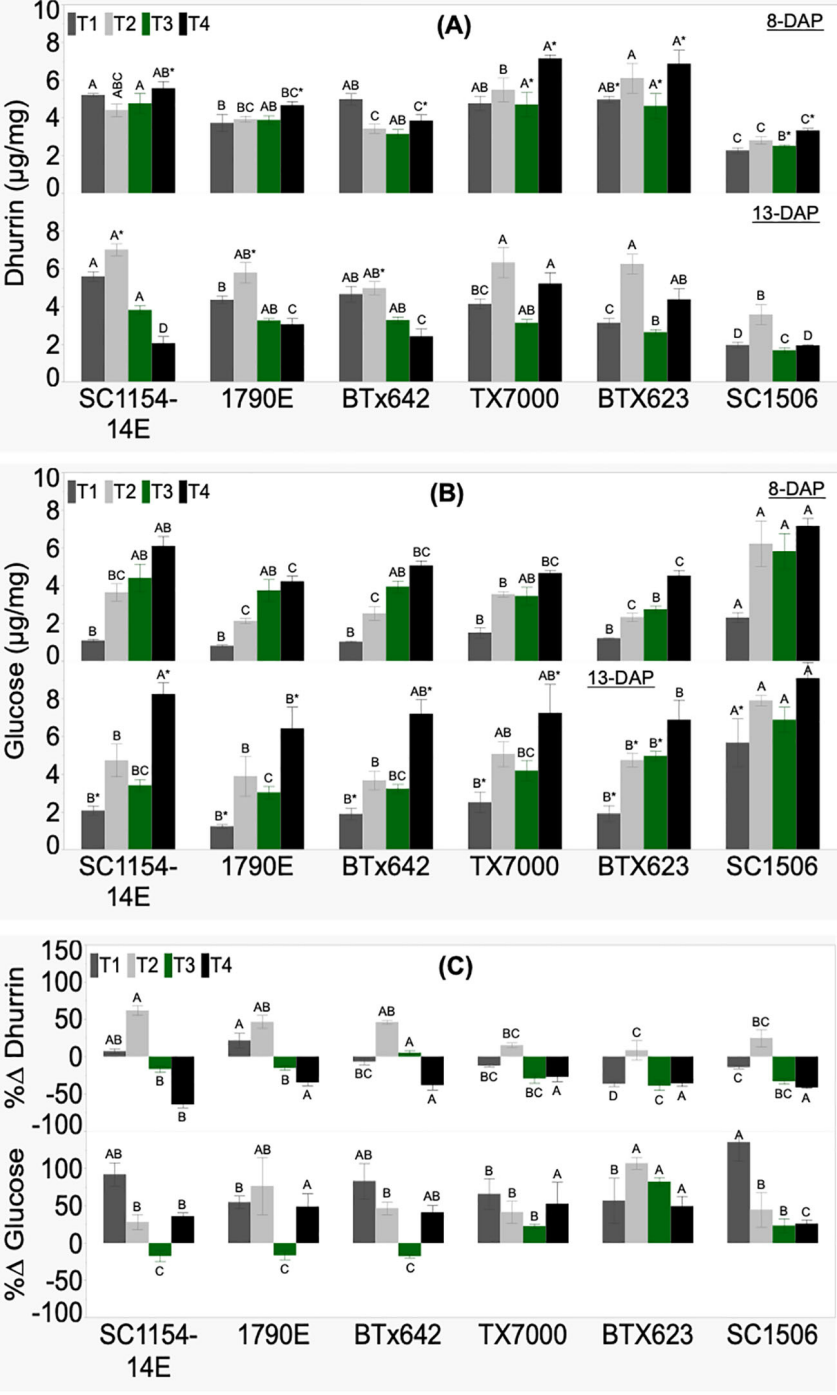
Genotypic variation of **(A)** dhurrin concentration, **(B)** glucose concentration, and **(C)** the percent change (%Δ) in each genotype from extractions of sorghum shoot tissue of seedlings at 8 and 13 DAP (days after planting) and quantified by HPLC analysis. Seedlings were grown in four substrate-fertility treatments (T1, T2, T3, and T4). Bars with different letters A, B, C for similar treatment at a given DAP are significantly different. An asterisk (*) on a letter indicates a significant difference between DAP for the given genotype under the specific treatment. High-dhurrin genotypes: SC1154-14E, 1790E, BTx642; Low-dhurrin genotypes: TX7000, BTx623, SC1506.

Genotypic variability in seedling dhurrin and glucose concentrations, and their respective %Δ was significant across treatments (**Figure 4**). There was no clear trend in seedling dhurrin concentration from high-to low-dhurrin level genotypes at 8 DAP irrespective of treatments (**Figure 4A**). At 13 DAP, dhurrin biosynthesis responded variably to nitrogen availability. The T1 (soil+N) treatment showed high-dhurrin genotypes to have significantly more dhurrin than their low counterparts, except for TX7000. Interestingly, the T2 (soil-N) and the T3 (sand+N) treatments did not show significant differences in dhurrin content between the high- and low-dhurrin genotypes, except in T3 where SC1506 had significantly lower dhurrin content than the other genotypes. Surprisingly, the T4 (sand-N) treatment showed the high-dhurrin genotypes to have lower dhurrin content than the low-dhurrin genotypes, except for SC-1506, which had similar levels as the high-dhurrin genotypes. All genotypes showed a significant decrease in dhurrin concentration from 8 to 13 DAP in sand-N (T4) only. Change in dhurrin content from 8 to 13 DAP under soil+N (T1) was insignificant across genotypes except in BTX623 where a significant decrease was observed. While high-dhurrin genotypes showed a pronounced and significant increase in dhurrin content from 8 to 13 DAP in soil-N (T2), the change in dhurrin content for low-dhurrin genotypes remained only slight and non-significant. In contrast, low-dhurrin genotypes showed a higher and significant reduction in dhurrin content from 8 to 13 DAP in sand+N (T3), while the change in dhurrin content for high-dhurrin genotypes was minute and non-significant. Percent change in dhurrin (%ΔDhurrin) was significantly different across genotypes irrespective of treatment (**Figure 4C**). Only in the T2 treatment were percent changes positive and significantly higher for all high-dhurrin genotypes than all low-dhurrin genotypes. All genotypes showed a negative %ΔDhurrin concentration under T4.

The glucose concentration of 8-DAP seedlings was significantly different between genotypes irrespective of treatment (**Figure 4B**). Additionally, there was no specific trend in glucose content at 8 DAP from high-to low-dhurrin genotypes, however, SC1506 was consistently and significantly higher than the other genotypes, particularly in the soil+N (T1) and soil-N (T2) treatments. At 13 DAP, no specific trend was observed for glucose concentration from high-to low-dhurrin genotypes. However, SC1506 maintained a significantly higher glucose concentration than the other genotypes under T1 and T3 (sand+N) treatments.

All genotypes showed a statistically significant increase in glucose concentration from 8 to 13 DAP in T1 and non-significant changes in T2 and T3 (except BTx623, statistically significant increase). All high-dhurrin genotypes significantly increased their glucose content from 8 to 13 DAP in T4, while low-dhurrin genotypes had non-significant changes (except for TX7000, which had an increase). Percent change in glucose content (%ΔGlucose) was positive and significantly different across genotypes under treatments T1, T2, and T4 (**Figure 4C**). Under T3, all low-dhurrin genotypes showed positive and significantly higher %ΔGlucose compared to the high-dhurrin genotypes, whose values were negative and significantly lower.

#### 3.2.2 Fructose and sucrose content

**Figure 5:**
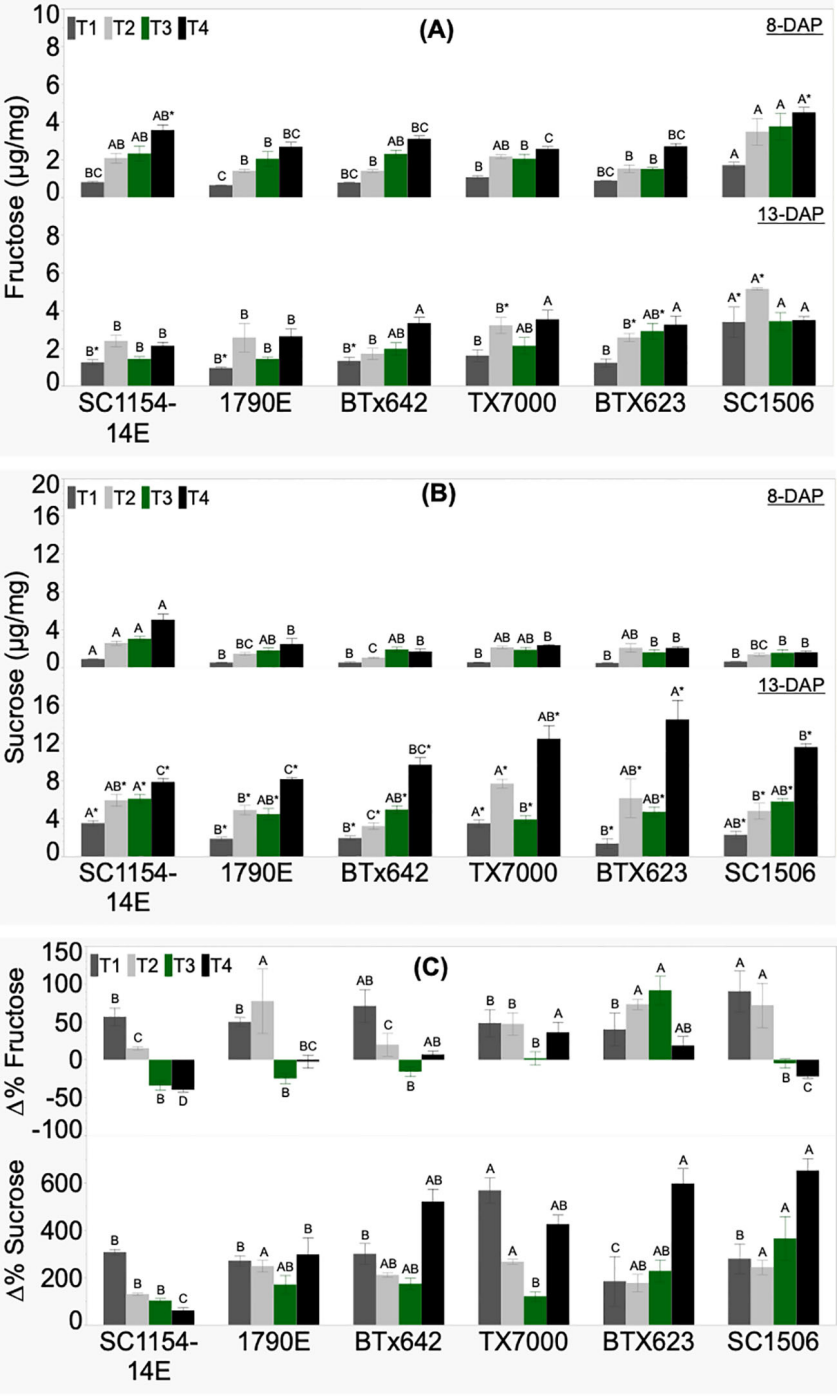
Genotypic variation of **(A)** fructose concentration, **(B)** sucrose concentration, and **(C)** the percent change (%Δ) in each genotype from extractions of sorghum shoot tissue of seedlings at 8 and 13 DAP (days after planting) and quantified by HPLC analysis. Seedlings were grown in four substrate-fertility treatments (T1, T2, T3, and T4). Bars with different letters A, B, C for similar treatment at a given DAP are significantly different. An asterisk (*) on a letter indicates a significant difference between DAP for the given genotype under the specific treatment. High-dhurrin genotypes: SC1154-14E, 1790E, BTx642; Low-dhurrin genotypes: TX7000, BTx623, SC1506.

Genotypic variability in seedling fructose and sucrose concentration, and their respective %Δ was significant across treatments (**Figure 5**). Fructose content at 8 DAP did not show a clear trend from high-to low-dhurrin genotypes irrespective of treatment (**Figure 5A**). However, SC1506 showed consistently high fructose content across treatments, and this was significantly higher under the T1 (soil+N) treatment. Fructose content at 13 DAP also showed no clear trend between the high- and low-dhurrin genotypes. Once again, SC1506 maintained a high fructose content across treatments, and was significantly different than the other genotypes under T1 (soil+N) and T2 (soil-N) treatments. All high-dhurrin genotypes showed a significant increase in fructose content from 8 to 13 DAP only under T1, while the change in low-dhurrin genotypes (except for SC1506, which had an increase) was non-significant. While all low-dhurrin genotypes showed a significant increase in fructose content from 8 to 13 DAP under T2, no significant change was observed in high-dhurrin genotypes. Fructose content in all genotypes (except for BTx623, which had an increase) was not affected by seedling age under T3. SC1506 fructose content was significantly higher compared to the other genotypes under T1 and T2 treatments at 13 DAP, and T1 treatment at 8 DAP. Percent change in fructose content (%ΔFructose) in high-dhurrin genotypes was negative and significantly lower than in low-dhurrin genotypes, except for SC1506, only in T3 (**Figure 5C**).

There was no clear difference in sucrose content between the high- and low-dhurrin genotypes at 8 DAP irrespective of treatments (**Figure 5B**). However, SC1154-14E had significantly higher sucrose contents under T1 and T4 than other genotypes. At 13 DAP, no defined trend in sucrose content was observed between the high- and low-dhurrin genotypes in T1, T2, and T3. However, under T4 (sand-N) treatment, values for sucrose content in the low-dhurrin genotypes were significantly higher than in the high-dhurrin genotypes. Sucrose content significantly increased with seedling age in all genotypes, irrespective of treatment. The percent change in sucrose content (%ΔSucrose) was positive and significantly different across genotypes irrespective of treatments, with no specific trend between the high- and low-dhurrin genotypes (**Figure 5C**).

#### 3.2.3 Oligosaccharide fructans signal peak

**Figure 6:**
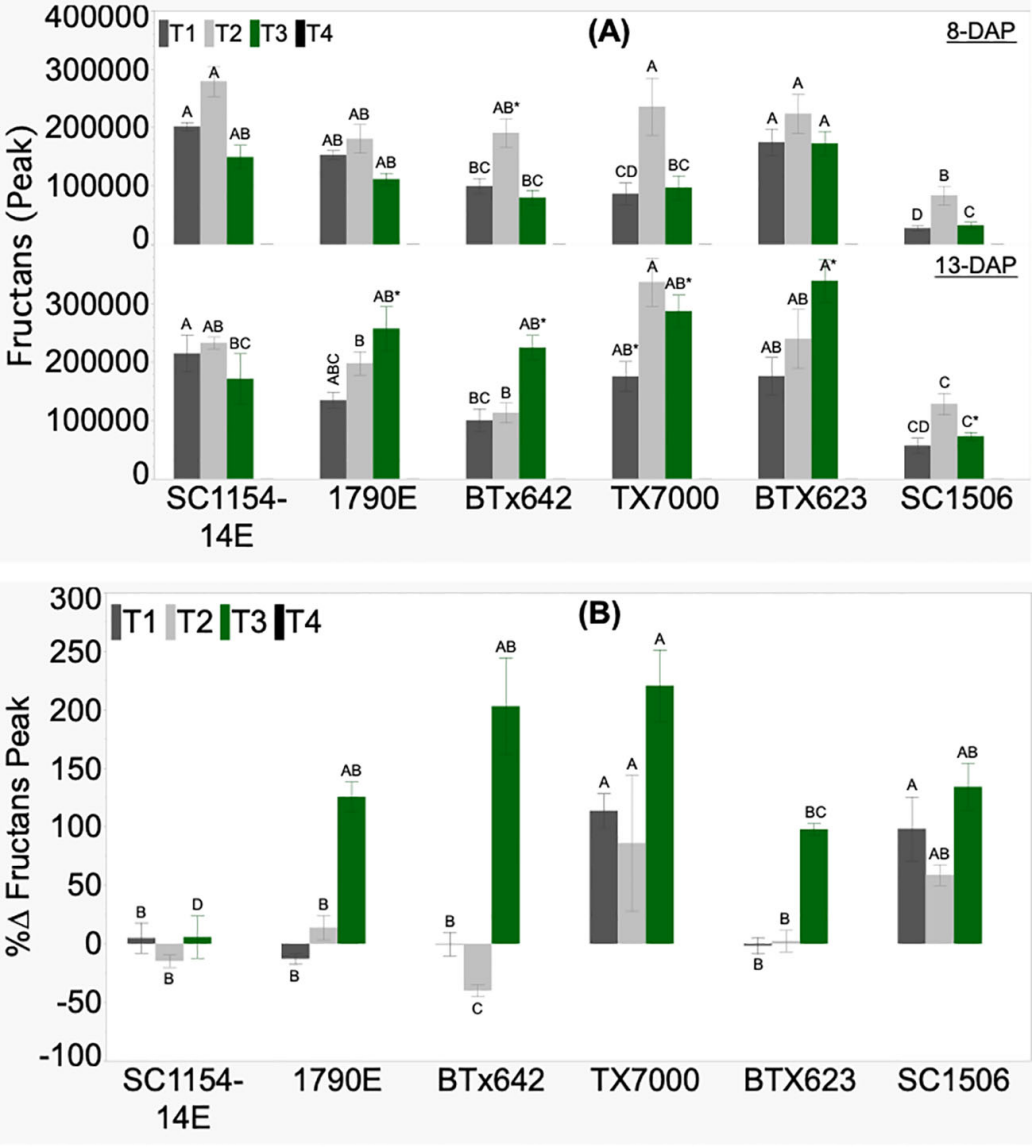
Genotypic variation of **(A)** fructans signal and **(B)** the percent change (%Δ) in each genotype from extractions of sorghum shoot tissue of seedlings at 8 and 13 DAP (days after planting) and quantified by HPLC analysis. Seedlings were grown in four substrate-fertility treatments (T1, T2, T3, and T4). Bars with different letters A, B, C for similar treatment at a given DAP are significantly different. An asterisk (*) on a letter indicates a significant difference between DAP for the given genotype under the specific treatment. High-dhurrin genotypes: SC1154-14E, 1790E, BTx642; Low-dhurrin genotypes: TX7000, BTx623, SC1506.

Relative fructans levels, based on raw signal peak values, did not show any defined trend between the high- and low-dhurrin genotypes, irrespective of seedling age and treatments (**Figure 6A**).

No fructans signal peak was detected across all genotypes in the sand-N water (T4) treatment. SC1506 showed consistently lower relative fructans levels across treatments on both days after planting. Only under sand+N (T3) did all the genotypes, except SC1154-14E, show a significantly higher fructans signal peak at 13 than at 8 DAP. All genotypes showed no significant change in fructans peak from 8 to 13 DAP under soil+N (T1) (except for TX7000, which had an increase) and soil-N (T2). The percent change in fructans signal peak (%ΔFructans signal peak) varied between genotypes across treatments (**Figure 6B**). All genotypes had positive percent change in fructans signal peak only in T3, with no defined trend observed between the high- and low-dhurrin genotypes.

#### 3.2.4 Relationships between dhurrin, sugars, and seedling growth

In the complete absence of any source of nitrogen, as in the sand-N treatment (T4), Pearson’s correlation matrix (**Table 4**) revealed a negative relationship between dhurrin and seedling growth parameters (FSW and FSL). The significance of these relationships increased with seedling age as r = -0.12 to r = -0.43 for FSW, and r = -0.47 to r = -0.56 for FSL at 8 to 13-DAP, respectively. Dhurrin showed negative or insignificant relationships to both monosaccharide soluble sugars (glucose and fructose) under all treatments, irrespective of seedling age, except at 13 DAP in T4, where the relationship with glucose (r = 0.26) and fructose (r = 0.61) was positive. The relationships between dhurrin and complex sugars (sucrose and fructans) were positive irrespective of seedling age and treatment, except in T4, where no fructans peak was detected irrespective of seedling age.

**Table 4:** Pearson’s correlation matrix between dhurrin (Dhu), growth parameters (FSW; fresh shoot weight and FSL; fresh shoot length), sugars (Glu; glucose, Fru; fructose, Suc; sucrose, and Ftn; fructans), and hundred grain weight (HGW) for sorghum seedling at 8 and 13 days after planting in different simulated substrate-fertility treatments.

Correlation variables	T1 (soil+N)		T2 (soil-N)		T3 (sand+N)		T4 (sand-N)	
	8 DAP	13 DAP	8 DAP	13 DAP	8 DAPS	13 DAP	8 DAP	13 DAP
Dhu-FSW	0.63	0.96	0.26	0.45	0.61	0.83	**-0.12**	**-0.43**
Dhu-FSL	0.57	0.74	-0.12	–	0.44	0.69	**-0.47**	**-0.56**
Dhu-HGW	0.69	0.86	0.27	0.63	0.50	0.86	0.33	–
Dhu-Glu	-0.33	-0.35	–	–	–	-0.53	-0.22	**0.26**
Dhu-Fru	-0.47	-0.35	–	–	–	-0.38	-0.33	**0.61**
Dhu-Suc	0.24	0.51	0.75	0.67	0.63	0.22	0.36	0.72
Dhu-Ftn	0.69	0.67	0.75	0.79	0.86	0.49	na	na
FSW-Glu	-0.58	-0.40	-0.27	-0.31	–	-0.44	–	**0.31**
FSW-Fru	-0.67	-0.38	-0.30	-0.41	-0.15	-0.19	0.12	-0.20
FSW-Suc	0.30	0.44	0.31	–	0.53	–	0.58	**-0.52**
FSW-Ftn	0.80	0.70	0.70	–	0.68	0.67	na	na
FSW-HGW	0.79	0.81	0.74	0.61	0.62	0.53	0.62	–
FSL-Glu	-0.27	–	–	–	–	-0.25	**0.39**	**0.38**
FSL-Fru	-0.36	–	–	–	–	–	0.46	–
FSL-Suc	0.20	0.39	0.12	–	0.35	0.24	0.45	**-0.43**
FSL-Ftn	0.51	0.58	0.48	**-0.23**	0.33	0.40	na	na
FSL-HGW	0.48	0.42	0.37	0.27	0.28	0.32	0.28	–
FSL-FSW	0.82	0.74	0.81	0.81	0.79	0.89	0.87	0.84

T1 is potting mix irrigated with N-fertilizer (soil+N), T2 is potting mix irrigated with deionized water (soil-N), T3 is play sand irrigated with N-fertilizer (sand+N), and T4 is play sand irrigated with deionized water (sand-N). “- “indicates the relationship was non-significant, and “na” means a fructans (Ftn) peak was not detected. Values in bold represent correlations that were significant at p<0.05.

Both fresh shoot weight and length showed a positive relationship to glucose (FSW; r = 0.31 and FSL; r = 0.38) and a negative relationship to sucrose (FSW; r = -0.52 and FSL; r = -0.43) only in T4. Both growth parameters showed positive relationships to fructans signal peak for treatments T1, T2, and T3, irrespective of seedling age. Hundred grain weight (HGW) consistently showed a positive relationship with dhurrin content and growth parameters, except at 13 DAP in the sand-N (T4) treatment, where the relationships were non-significant.

#### 3.2.5 Unique contributions of dhurrin and sugars to seedling growth

A multiple linear regression analysis was performed to characterize the unique contributions (*β*) of dhurrin and sugar contents, respectively, to the observed variance in seedling growth parameters (**Table 5**). Dhurrin concentration was only statistically significant in explaining observed variations in shoot weight on both days after planting in the sand-N (T4) treatment (**Table 5a**). Glucose had a statistically significant contribution to FSW on both DAP in T1, at 13 DAP in T3, and at 8 DAP in T4. The contribution of fructose to FSW was only statistically significant on both DAP in T4. The unique contribution of sucrose to FSW was statistically significant at 13 DAP in T4, whereas the fructans contribution was only statistically significant at 13 DAP in T3. HGW was not a statistically significant contributor to FSW at both DAP in T1 and T4.

**Table 5:** Beta-values (*β*) from multiple linear regression model indicating the unique contribution of dhurrin, glucose, fructose, sucrose, fructans, and hundred grain weight in explaining observed variances in fresh shoot weight (a) and fresh shoot length (b) of sorghum seedlings seedling at 8-and 13-days after planting in different simulated substrate-fertility treatments.

(a) Fresh shoot weight (mg)								
	T1		T2		T3		T4	
	8 DAP	13 DAP	8 DAP	13 DAP	8 DAP	13 DAP	8 DAP	13 DAP
Dhurrin	-0.06	-0.07	-0.28	-0.29	**-0.39**	-0.14	**-0.56**	**-0.46** *^β^*
Glucose	**-0.47**	**-1.98**	-0.37	-0.23	0.05	**-1.07**	**-0.89**	0.11
Fructose	-0.04	1.69	0.08	-0.15	-0.02	0.49	**0.82**	**-0.40**
Sucrose	0.05	0.11	-0.19	-0.24	0.10	-0.14	0.13	**-0.32**
Fructans	0.23	0.13	0.10	-0.05	0.37	**0.37**	na	na
HGW	0.33	0.58	**0.67**	**0.62**	**0.48**	0.25	0.71	0.17
*R-Squared*	*0.80*	*0.71*	*0.70*	*0.69*	*0.53*	*0.71*	*0.78*	*0.79*
(b) Fresh shoot length (cm)								
Dhurrin	0.07	-0.43	**-0.47**	**-0.44**	-0.18	-0.30	**-0.69**	**-0.51**
Glucose	-0.37	-2.53	-0.38	-0.41	0.02	-1.62	**-0.84**	0.26
Fructose	-0.04	2.28	0.15	0.45	-0.12	1.14	**0.95**	**-0.48**
Sucrose	-0.01	0.06	-0.11	**-0.42**	-0.06	-0.15	**0.29**	**-0.37**
Fructans	0.00	-0.02	-0.09	-0.24	-0.05	0.13	na	na
HGW	0.32	0.61	0.46	**0.69**	**0.33**	0.32	**0.36**	**-0.39**
*R-Squared*	*0.34*	*0.27*	*0.39*	*0.59*	*0.10*	*0.45*	*0.78*	*0.75*

*^β^* The magnitude of the beta-value indicates the unique contribution by the variable (dhurrin, glucose, fructose, sucrose, or HGW) to explaining the dependent variable (fresh shoot weight or fresh shoot length), when all the variance explained by all other variables in the model is controlled for. Bold *β*-values indicate the unique contribution by the variable was significant. *R-squared* indicates how much of the variance in either fresh shoot weight or fresh shoot length is explained by the model. **“**na” means a fructans peak signal was not detected.

Dhurrin concentration was also a statistically significant contributor to observed variations in shoot length in both N-deficient treatments T2 and T4 (**Table 5b**). Glucose, fructose, and sucrose contributions to FSL were statistically significant only in T4, while the unique contribution of fructans to FSL levels was not statistically significant irrespective of treatment or seedling age. HGW was a statistically significant contributor to FSL under both DAP in T4 but not to FSW.

## 4 Discussion

Dhurrin has been shown to function as a metabolic N-turnover, N-transport, and N-storage secondary metabolite (Kongsawadworakul et al., 2009) in sorghum. As a metabolic N transporter, the endogenous dhurrin turnover pathway enables the sorghum plant to extract nitrogen and glucose deposited in dhurrin, for use in primary metabolism (Møller, 2010; Bjarnholt et al., 2018). Bjarnholt et al. (2018) helped elucidate this pathway via several metabolic experiments that showed that the breakdown of dhurrin was indeed occurring via an alternate recycling pathway, other than bioactivation/detoxification. This pathway has been proposed to be particularly active during grain maturation, and it has been shown through transcriptomics (Gleadow et al., 2021) that the genes proposed to be involved in dhurrin turnover/recycling are upregulated during this stage. Gleadow et al. (2021) proposed that the increased expression of dhurrin recycling genes near floral initiation, at anthesis, and during grain maturity could reflect an increased demand for nitrogen for seed set and filling.

However, reports on dhurrin’s role in germination and, particularly, early seedling growth have been contradictory. Using total cyanide-deficient mutants (*tcd1*), Montini et al. (2020) reported delayed germination. Blomstedt (2012); Blomstedt et al. (2018), and Sohail et al. (2020) also reported reduced early seedling growth, but Gruss et al. (2022) reported larger green plant area and dry weight of seedlings of *cyp79a1* BTx623 mutants, compared to the wild type. Both *tcd1* and *cyp79a1* mutants led to mutations in the dhurrin biosynthetic enzyme CYP79A1, resulting in the complete loss of dhurrin biosynthesis (Blomstedt, 2012; Tuinstra et al., 2016).

To evaluate the role of dhurrin as a possible nitrogen source in germination and early seedling growth under nitrogen demand, the current research subjected seedlings of non-mutant sorghum genotypes varying in dhurrin concentration (high and low, determined from the leaves of matured plants) in marginal (N-deficient) and non-marginal (N-available) growth conditions (play sand and potting soil with and without N fertilization). The contents of the secondary metabolite (dhurrin) and primary metabolites (glucose, fructose, sucrose, and fructans), and seedling growth characteristics (fresh shoot weight and length) were evaluated at 8 and 13 DAP (equivalent to 5 and 10 days after emergence; DAE).

### 4.1 Dhurrin may have a minimal effect on seedling growth from germination to 8 DAP

During early seedling growth, from germination to 8 DAP, except for genotype SC1506, which is a known ultra-low dhurrin genotype (Emendack et al., 2018), the concentration of dhurrin did not show any trends in differences between the high- and low-dhurrin genotypes, irrespective of the substrate-fertilization treatment (**Figure 4A**). A similar observation (**Figures 2A, B**) was made for 8-DAP FSL and FSW, suggesting that dhurrin, as a secondary metabolite, may have a minimal effect on growth from germination to 8 days after planting. This lack of difference in dhurrin concentration between the high- and low-dhurrin genotypes at early seedling stages of growth was also observed by Haskins et. al (1987). Using high- and low-cyanide potential genotypes, the authors showed that both sets of genotypes had similarly high hydrogen cyanide potential (HCN_p_) at the early seedling stage, but significantly higher HCN_p_ in favor of the high genotypes when mature leaves from field-grown plants were tested. That dhurrin may not have a large effect on seedling growth from germination to 8 DAP is also supported by other research findings. Newton et al. (1980) stated that, during early seedling growth in sorghum, the endosperm’s sugar content continuously depletes as dhurrin content increases.

At 8 DAP, the seedling is autotrophic with 75% of the endosperm depleted and dhurrin content at its maximum. At this stage, dhurrin may constitute up to 30% of the dry shoot weight of leaves and coleoptile of etiolated sorghum seedlings (Saunder and Conn, 1977; Halkier and Møller, 1989), and 6% of the dry shoot weight of young seedlings (Busk and Møller, 2002). The cyanide potential (HCN_p_) of sorghum seedlings has been shown to peak at 4 days after emergence and remain unchanged up to 8 days after emergence, irrespective of N fertilization (Haskins et al., 1987; Busk and Møller, 2002). Thus, in our experimental system, while seed endosperm (starch, protein, and lipids) was the main reserve of energy for germination (Yan et al., 2014; Carrera-Castano et al., 2024), the endosperm, and not dhurrin, may have also served as an energy resource for growth up to 8 DAP (equivalent 4 to 5 days after emergence). Reports have shown that, endosperm starch and lipids give rise to sugars during seed germination (Hill et al., 2003; Awatif and Alaaeldin, 2017), which are transported and required for growth (Mayer and Poljakoff-Mayber, 1975).

### 4.2 Seedling growth trends are related to dhurrin depletion

As the seedlings aged (8 to 13 DAP), we observed a statistically significant (*p<0.*05) decrease in dhurrin concentration with a concomitant and statistically significant *(p<0.*05) increase in glucose and sucrose concentrations across all genotypes, but *only* under complete N-deficient conditions (T4; sand irrigated with deionized water) (**Figures 4A, B**, **5B**). Nitrogen availability is important for seedling growth. Rivai et al. (2021) showed that BTx623 seedlings had a 51% reduction in dry weight when grown under N-limited conditions. Additionally, Blomstedt et al. (2018) also showed that N treatment was the most significant factor when comparing the growth of *tcd1, acdc1*, and BTx623 plants under three N treatment conditions. It is interesting to postulate that, considering the importance of N to growth combined with the complete lack of N in the T4 treatment, perhaps the endogenous alternate turnover pathway for dhurrin (**Figure 1**) was activated by the N stress being applied to provide a source of reduced nitrogen for growth. In this pathway, dhurrin is turned over through enzymatic actions (*Glutathione S-transeferase, Glutathione S-lyase*, and *Nitrilase heteromer NIT4*) to *p*-Hydroxyphenylacetic acid and NH_3_, with glucose as a primary by-product (Gleadow and Møller, 2014). The NH_3_ could serve as an N source for seedling growth while the simple soluble sugar glucose is transformed into its complex storage form, sucrose. The %ΔDhurrin from 8 to 13 DAP under N-deficient conditions (T4) was greater in the high-dhurrin genotypes compared to the low-dhurrin (except SC1506) genotypes (**Figure 4C**). Presumably, as the dhurrin may have been used as an N source to build biomass and length, the increase in growth parameters in T4 (**Figures 3A, B**) became greater for the high-dhurrin genotypes compared to the low-dhurrin (except SC1506) genotypes. This increase was more pronounced for fresh shoot weight, where all the high-dhurrin genotypes had higher %ΔFSW than all the low-dhurrin (except SC1506) genotypes. While there was a statistically significant increase in shoot length from 8 to 13 DAP, no trend was observed from the high- to low-dhurrin genotypes. These observed increases in growth parameters with decreases in dhurrin concentration, accompanied by increases in glucose and sucrose concentration across all genotypes from 8 to 13 DAP, under complete N-deficient conditions, provided evidence that NH_3_ from dhurrin turnover may have been an N source for seedling growth, especially under N demand as elicited by N deficiency. Emendack et al. (2017) reported that under simulated marginal soil conditions, seedlings of stay-green genotypes, characterized by their high dhurrin concentration (determined from the leaves of mature plants; Hayes et al., 2015), showed better seedling growth than their senescent (non-stay-green) counterparts.

The connection between dhurrin concentration and seedling growth, occurring across the five days between sampling (8 and 13 DAP), was also observed in the T2 treatment (soil-N). Interestingly, under a progressively depleting N stress condition (T2; potting soil irrigated with deionized water), where N availability declined over time due to seedling uptake with no external replenishment, we observed a statistically significant (*p<0.05*) increase in dhurrin concentration (**Figure 4A**) across seedling age in the high-dhurrin genotypes, but *not* in the low-dhurrin genotypes. Predictably, an obvious trend was also seen in %ΔDhurrin (**Figure 4C**) from high- to low-dhurrin genotypes, which was markedly higher in the high-dhurrin genotypes. This response may suggest that the seedlings of the high-dhurrin genotypes perhaps responded more acutely to the progressive N stress by building dhurrin pools from which they could maintain growth as any residual nitrogen in the potting mix was depleted. This response provided more evidence of the possible connection between dhurrin, as an N source, and seedling growth under N-stress conditions. Unlike the complete N-deficient condition (T4) where all sugar pools had significant statistical changes (*p<0.05*) in most of the genotypes across the two seedling ages, the T2 treatment showed only sucrose to have a statically significant (*p*<0.05) increase in concentration in most genotypes across seedling age (**Figures 4B**, **5A, B**). However, there was no obvious trend in sucrose increase from the high- to low-dhurrin genotypes. Fresh shoot biomass also increased (*p<0.05*) across genotypes in the T2 treatment, with the high-dhurrin genotypes also showing larger increases in %ΔFSW on average (**Figure 3C**) compared to the low-dhurrin genotypes. Likewise, fresh shoot length (**Figure 3B**) showed a statistical increase (*p<0.05*) in all the high-dhurrin genotypes but had non-statistically significant changes in the low-dhurrin genotypes, except for SC1506, which showed a statistically significant increase (*p<0.05*).

While dhurrin content and growth parameters showed a connection in the T2 and T4 treatments when N was limited, we sought to determine whether dhurrin was correlated to and predictive of seedling growth under extreme N-stress conditions, such as in treatment T4. Pearson’s correlation analysis (**Table 4**) showed a statistically significant (*p<0.05*) negative correlation between dhurrin concentration and FSW (-0.43) and FSL (-0.56) at 13 DAP under the T4 treatment condition (sand-N) only. Multiple linear regression analysis (**Table 5**) furthered this result with dhurrin concentration, showing a considerable and statistically significant (*p<0.05*) contribution to predicting FSW (*β* = -0.56 and -0.46) and FSL (*β* = -0.69 and -0.51) at 8 and 13 DAP, respectively, under the T4 treatment. Taken together, our results provide evidence that dhurrin could serve as a possible N source for maintaining early seedling growth in sorghum under N-deficient conditions.

### 4.3 Dhurrin content alone may not be the only contributing factor in maintaining early seedling growth under N-deficiency

Our results showed a statistically significant association and provided evidence of a connection between dhurrin content and sustained seedling growth under N-stress conditions. This response was more pronounced in the high-dhurrin genotypes; however, it was not completely absent in the low-dhurrin genotypes. The low-dhurrin genotypes did exhibit a slight decrease in dhurrin content and a slight increase in FSW under T4 conditions (sand-N) (**Figures 3A**, **4A**). The differential seedling response among the genotypes of dhurrin depletion with continued growth under N deficiency could not have been due to dhurrin concentration alone. With the exception of the ultra-low dhurrin genotype, SC1506, the dhurrin levels between high and low-dhurrin genotypes did not differ significantly at 8 DAP before N stress intensified towards 13 DAP. This suggests that maybe there is a genetic and/or metabolic control mechanism that activates the dhurrin turnover pathway under N deficiency, and which perhaps is more highly repressed or absent in some genotypes at the seedling stage of development. Gleadow et al. (2021) provided transcriptomic evidence that dhurrin biosynthetic *and* recycling genes had increased expression levels in imbibed seeds and in leaf blades of BTx623 at 8 days after emergence. It is interesting to postulate that perhaps under N deficiency, the expression of these genes is modulated differentially.

In this respect, the metabolic response of SC1506, the ultra-low dhurrin entry in our genotypic panel, to N deficiency, provides an interesting evidential exhibit. SC1506, although lower in dhurrin content than the other low-dhurrin genotypes at 8 DAP (**Figure 4A**), showed a statistically significant (*p<0.*05) increase in FSW and statistically significant (*p<0.*05) decrease in dhurrin content under N-deficiency (T4) conditions that was similar to the high-dhurrin genotypes. In fact, SC1506 had the highest %ΔFSW and %ΔFSL among all the genotypes tested under the T4 treatment (**Figure 3C**). These results suggest that SC1506, although lower in dhurrin concentration, possibly has an active mechanism that allows it to catabolize dhurrin for use as an N-source to maintain FSW and FSL under N-deficiency. It would be interesting to determine how the *tcd1* and *acdc1* mutants would respond to complete N deficiency at the seedling stage. Further studies to determine how the dhurrin recycling pathway is modulated under N deficiency at the seedling stage would provide valuable insight into dhurrin metabolism.

### 4.4 Complications of dhurrin-free mutants for early seedling growth and establishment

Dhurrin, as a cyanogenic glucoside, is a multi-functional secondary metabolite credited for its insect repellent, grazing defense, nitrogen storage, and osmoprotectant properties. Dhurrin catabolism by the enzyme *dhurrinase*, leads to the production of HCN, commonly known as prussic acid, which is poisonous and can lead to death in cattle when grazing on young leaves of sorghum plants subjected to drought, freezing, or high N fertilization. Breeding for low cyanide or acyanogenic potential for cattle grazing and forage has become a major priority to increase the economic value of sorghum as a feed crop. However, recent contrasting reports on the reduced agronomic performance of acyanogenic mutants (*tcd1* or *cyp79a1*) as it relates to stand establishment and early seedling growth in the fields call for research on the role of dhurrin as an N source for early seedling growth in sorghum. Our report has provided positive evidential basis for a link between dhurrin content and early seedling growth, with dhurrin possibly acting as an N-source under N-deficient conditions, conceivably through the catabolic alternate recycling pathway, which produces NH_3_ as a by-product. Additionally, our findings also propose that, if in fact the turnover pathway is involved under N deficiency, not all sorghum genotypes activate this turnover pathway to the same degree.

One of the genotypes in our panel was BTx623, which has been used as a main genetic background to produce acyanogenic mutants (Tuinstra et al., 2016). In our study, BTx623 showed sufficient levels of dhurrin at 8 DAP, similar to four other genotypes in our panel. However, when exposed to N deficiency, its growth response was reduced on a percentage basis as compared to the high-dhurrin genotypes (SC1154, 1790E, and BTx642) and the ultra-low dhurrin genotype SC1506. Thus, by using acyanogenic mutants that have the BTx623 background as breeding sources for acyanogenic sorghum forage, it is plausible that the complete loss of dhurrin as a potential N source may be a reason for the slower growth and germination problems previously reported in these mutants. Perhaps a better source for breeding low-cyanogenic sorghum would be genotypes such as SC1506 that have sufficient (although still low) dhurrin at the early seedling stage, along with continued growth even under N-deficient conditions.

## 5 Conclusion

Using non-mutant genotypes with variable dhurrin levels, as determined from the leaves of mature plants, this research reports evidence that dhurrin is a potential source for nitrogen in early seedling growth, when plants are subjected to N-deficient or N-limited environments.

This finding is critical, considering that the cultivation of sorghum in most regions, particularly in sub-Saharan Africa and Asia, where grain sorghum is a staple, is usually in low-input environments, where fertilization is minimal at best and lacking at worst.

While acyanogenic sorghum will be advantageous for expanding sorghum’s economic value, acyanogenic mutants have been shown to have slower growth. As such, the use of dhurrin knock-out mutants, with plausible background mutations, may not be advisable, given the potential vital role of dhurrin, as an N turnover source, could have in early seedling growth, especially under low nitrogen input or marginal conditions. Furthermore, the current findings, showing seedlings of high-dhurrin genotypes outperforming low-dhurrin genotypes in early seedling growth (fresh shoot biomass and length) under N-deficient conditions, are vital information for breeding programs in low-input sorghum cultivating regions.

## Data Availability

The original contributions presented in the study are included in the article/supplementary material. Further inquiries can be directed to the corresponding author.
